# Rational Resampling Ratio as Enhancement to Shaft Imbalance Detection

**DOI:** 10.3390/s23031719

**Published:** 2023-02-03

**Authors:** Adam Jablonski

**Affiliations:** Department of Robotics and Mechatronics, Faculty of Mechanical Engineering and Robotics, AGH University of Science and Technology, 30-059 Krakow, Poland; ajab@agh.edu.pl

**Keywords:** imbalance detection, resampling, rational transmission ratio, machine diagnostics

## Abstract

Trend analysis is one of the most powerful techniques for monitoring the technical condition of individual mechanical components of rotating machinery. It is based on extraction of characteristic signal components according to kinetostatic configuration of the machine drivetrain. It has been used for decades and is well-understood. However, classical trend analysis is based on some assumptions which have resulted from the limited computational power of embedded systems years ago. This paper tries to answer a question on whether the assumption of a single signal resampling path for calculation of signal components generated by shafts with rational transmission ratio is valid. The study was conducted using an extensive imbalance test on a medium-power test rig. The paper originally demonstrates that application of an advanced resampling algorithm does not significantly influence the overall trend increase, but it is of utmost importance when trend variance is of interest.

## 1. Introduction

**Figure 1:**
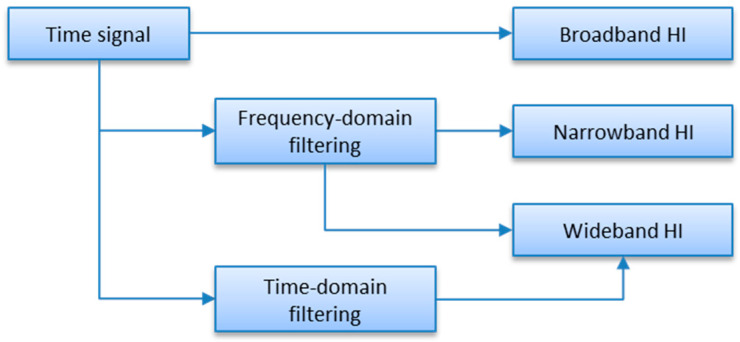
Calculation paths of scalar machine health indicators.

Continuous condition monitoring of rotating machinery is typically based on trend analysis, which relies on tracking of scalar indicators over time [1]. These scalar indicators, also called “health indicators” (HI) or “diagnostic features”, must therefore be predefined, as a part of CMS (Condition Monitoring System) configuration. Typical classification of scalar indicators differentiates among broadband indicators, wideband indicators, and narrowband indicators. Broadband indicators are these, which cover an entire signal spectral range. They include, for instance, peak-to-peak (PP) value, or zero-peak (ZP) value, root-mean-square (RMS) value, crest factor, and kurtosis. Narrowband health indicators are calculated from selected frequency/order bands, that is, from narrowband signals. Narrowband signals are obtained by filtering input signals with bandpass filters. These filters are applied either in the time domain or in the frequency/order domain; however, when relatively narrow bandpass filters are considered, the frequency/order domain filters are in favor. Such filtering boils down to selecting consecutive spectral indexes. Narrowband trends are widely used to track phase-locked components, like shaft components, gearbox components and rolling element-bearing components (local faults). Wideband indicators are such, which by an arbitrary decision cover a wide bandwidth. A typical example of a wideband indicator is velocity RMS (VRMS), because according to the ISO 20,816 standard, it is calculated from a velocity signal filtered either in the band 10–1000 Hz or 2–1000 Hz. This basic concept is illustrated in Figure 1.

**Figure 2:**
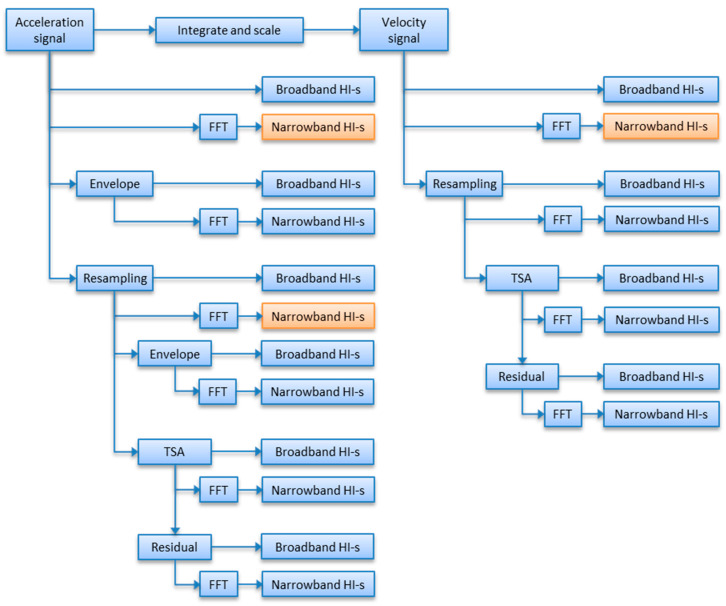
Placement of analyzed scalar machine lar HI-s (health indicators).

The object of the presented research was to quantitatively assess the capability of detection of shaft imbalance of different narrowband health indicators, with special attention given to order-domain indicators calculated with different resampling ratios. Thus, the paper tries to answer the question of whether it is justified to calculate multiple versions of angle-domain signals for drive trains with multiple transmission ratios and only one phase marker available. For reference purposes, the presented analysis also shows trends of corresponding selected broadband and narrowband health indicators for the same imbalance fault development. The flowchart of analyzed indicators is given in Figure 2. The presented paths of calculation of scalar health indicators are included in the paper to map the scope of the paper (orange blocks) on the entire structure of indicators used in condition monitoring. As shown, broadband health indicators are calculated from a raw acceleration signal, its envelope, resampled signal, envelope of the sampled signal, time-synchronous-averaged signals (TSA), introduced in the Ref. [2], later described in detail in the Ref. [3] or the residual part. TSA has also been successfully applied to rolling element bearings [4,5,6] and gearboxes [7]. Advanced techniques of shaft fault detection methods are given in the Ref. [8]. In the case of velocity signals, this group includes all but envelope (high frequency analysis) signals. At the same time, according to Figure 2, narrowband health indicators are calculated from spectral representations of various listed signal transformations. Note that only VRMS is analyzed within the velocity physical domain, because point-wise narrowband analysis across acceleration and velocity domain by definition yields analytically equal results (as both domains are related by scaling factor 1/2 × pi × frequency).

Note that each node in the tree in Figure 2 reflects more complex calculations, which is a major challenge for embedded systems, not only due to computational capabilities but also in terms of power consumption. As a result, industrial systems are designed to perform as little calculations as possible to meet requirements under the constraints of particular applications. The current paper illustrates the potential benefits and drawbacks of extra signal processing resampling algorithms when detection and identification of shaft imbalance state is of interest.

The main object of the paper is to present the influence of different resampling ratios on the fault detection of shaft imbalance of rotating machinery based on trend analysis of narrowband components, translated by CMS into scalar health indicators. For this reason, this section discusses what resampling is in general, what the resampling relative ratio is, and how narrowband components can be calculated. The paper is organized as follows: Section 2 discusses the current findings in the subject with an emphasis on resampling designed for rational transmission ratios. Section 3 describes the mechanical test rig, plus gives details on how imbalance was introduced and how it is identified. Section 4 presents corresponding trend analysis along with quantitative assessment based on two statistical criteria. Section 5 briefly discusses conclusions from the point of view of possible enhancement of trend analysis with the method presented in the paper.

## 2. Theoretical Background

### 2.1. Resampling

**Figure 3:**
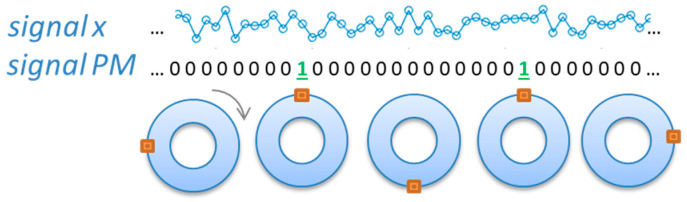
Idea of the 1-per-rotation PM signal.

Resampling is a mathematical operation-transforming signal from the time domain to angle domain. Typically, it is used to reduce the negative influence of the variable machine speed on identification of spectral components of the analyzed signal. Let us consider a recorded vibration signal “x” and a phase marker signal PM recorded in parallel. The PM signal has a form [0 … 0 0 1 0 0 …. 0 0 1 0 … 0], where value “1” represents a consecutive shaft rotation. This idea is presented in Figure 3.

**Figure 4:**
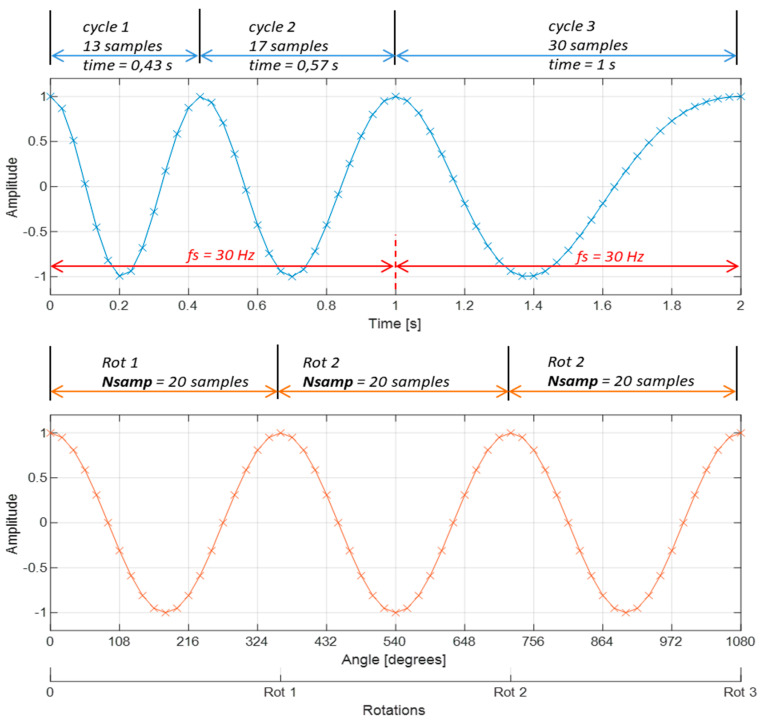
Top: signal sampled with constant sampling frequency, bottom: signal sampled with a constant number of samples per rotation.

As a result of resampling, the original signal, which was recorded with a constant number of samples in unit of time, like 25,000 samples per second (25 kHz sampling frequency), is converted to a signal with a constant (like 20) number of points per rotation. Figure 4 illustrates this process for a signal with decreasing frequency. This example could be referred to a rotating machinery, the speed of which decreases monotonically.

**Figure 5:**
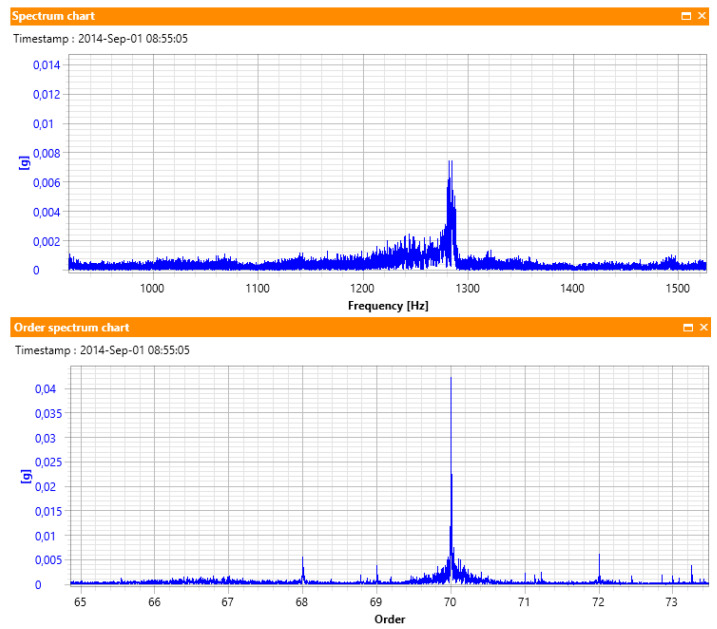
Demonstration of resampling ability to reduce spectral smearing.

From a visual interpretation of Figure 4, it is clear that in the original signal, the first rotation is faster than the first rotation in the resampled signal, while in the case of the last (third) rotation, it is the opposite. This impression is correct because the higher the speed of the shaft, the number of samples per rotation decreases, for a constant sampling frequency in the time domain. In the resultant resampled signal, speed variations are not visible, and the horizontal axis represents the angle, not time. For the constant sampling frequency *fs*, the first rotation has 13 samples, the next one has 17 samples, and the third one has 30 samples. Thus, the average number of samples per rotation is 20, and this number was used to create Figure 4. Most importantly, each shaft rotation has the same number of samples, *Nsamp*. In order to show the practical meaning of resampling, Figure 5 illustrates examples of spectra from the same vibration signal, namely the full-resolution vibration spectrum and its resampled version.

Figure 5 illustrates two spectra from a vibration signal recorded from a machinery working with variable speed. During the acquisition, machine speed varied in the range 1045–1100 RPM. The gear meshing component, which is equal to order 70, varied from 1219 Hz to 1283 in this time window, which makes it smeared in the regular spectrum, but clearly identifiable in the order spectrum, which is calculated from the resampled signal.

The concept of resampling has been described widely in the literature. A detailed analysis of the influence of interpolation function on the shape of spectral components is given in the Ref. [9]. Practical implementation of resampling to an industrial application is given in the Ref. [10]. Some researchers show how signals could be resampled from a recovered PM signal [11,12]. Detailed mathematical analytics on the resampling techniques are given in the Refs. [13,14,15]. Application of resampling to non-stationary signals is given in the Ref. [16].

### 2.2. Resampling Ratio

In a typical scenario, the phase marker sensor is located on the fastest input shaft of the drivetrain (driving end), in order to keep the maximum possible accuracy of the measurement. However, for non-integer transmission ratios, this means that for every rotation of the input shaft, the output shaft (slower shaft) rotates a non-integer number of rotations. Thus, when the resampling process is done with respect to the input shaft, the potential imbalance component, located at an order equal to 1, enters the FFT (Fast Fourier Transform) algorithm with a complete integer number of cycles. However, in the drivetrain considered in the paper, the imbalance condition is experienced by a shaft, the ratio of which is expressed as a rational number with respect to the reference shaft. In this way, the output shaft imbalance component is located at an order equal to 23/67 (approximately 0.343). This means that when it enters the FFT algorithm with an incomplete number of cycles, it results in spectral smearing. One of the possible solutions to this problem is to resample the signal with respect to the output shaft, that is, as if the PM sensor was located on the output shaft. Mathematical details of this operation are given in the Ref. [17], with additional background given in the Refs. [18,19]. The idea of relating the resampling parameters to external signals is proposed in the Ref. [20]. The object of this paper is to quantitatively evaluate the potential benefit of such an operation from the point of view of popular trend-based condition monitoring.

### 2.3. Narrowband Component Extraction and Calculation Techniques

When a machine HI like “Shaft x1” is considered, its name does not reveal the calculation path. Such components could be calculated in the time domain, frequency domain, order domain, their envelope versions, and so forth. For constant-speed machinery, the imbalance component might refer to the frequency spectrum; however, in most cases, it refers to the order spectrum component. Next, on the basis of kinetostatic analysis, the spectra band is defined. Typically, this band corresponds to a 3% width of the analytically calculated imbalance spectral location [21,22]. In this way, a set of consecutive spectral amplitudes is determined. In the final step, a predefined mathematical calculation is applied to these sets of values. Typical calculations include maximum value, power, and root-mean-square (RMS).

## 3. Experiment Description

The research study described in the paper was performed on a test rig design to provide conditions close to industrial cases.

### 3.1. Mechanical Rig

**Figure 6:**
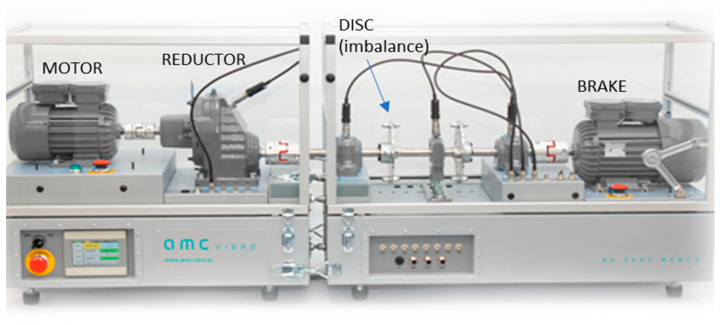
Test bench.

The test rig used in the experiment is illustrated in Figure 6. It is a drivetrain, which includes an input reference shaft driven by a 1.1 kW electrical motor, one-stage parallel gearbox, an output shaft, a set of rolling-element bearings, and a braking motor. The gearbox is a speed reductor with 23 teeth on the gear on the Input Shaft and 67 teeth on the gear on the output shaft, giving a total ratio of 23/67 = 0.34328. The bearings are generally spherical, with a double row roller 22,202 EK bearing. In order to introduce the gradually increasing imbalance condition, two round plates with multiple holes are installed on the output shaft.

**Figure 7:**
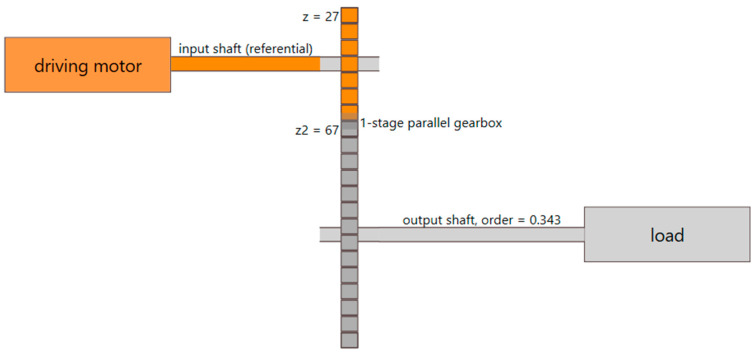
Kinetostatic diagram of the rotating machinery used in the experiment.

During the analyzed experiment, the test rig runs at 3000 RPM rotational speed (input shaft), and 50% nominal load, which is about 1.8 Nm. The kinetostatic configuration of the drive train is given in Figure 7.

The idea of the paper is to analyze a case where imbalance occurs to a different shaft than the one where the speed is measured, plus on such a shaft, for which the relative order is expressed by a rational number with respect to a reference shaft. As seen in Figure 7, the speed of the output shaft is related to the speed of the reference shaft by a factor of 23/67, which is a non-integer rational number.

### 3.2. State of Shaft Imbalance

**Figure 8:**
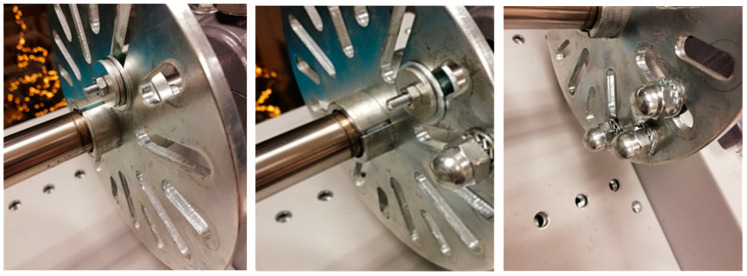
From the left: initial and two subsequent intermediate imbalance states.

The paper investigates development of the imbalance condition of the output shaft. The imbalance condition is introduced using a series of weights attached to one of the plates on the output shaft. Two types of weights are used, namely a smaller screw with a different number of washers, and from 1 to 6 larger screws. Figure 8 illustrates the selected initial stage, and two intermediate stages of the introduced imbalance state.

Additional weight was being introduced in a pseudo-monotonic manner in order to reach the impermissible level of vibrations according to ISO 20816, that is, when the velocity RMS in the range of 10 Hz to 1000 Hz reached ca. 11 mm/s.

### 3.3. Data Acquisition

Subsequent vibration signals were recorded after each imbalance state modification. A Monitran^®^ MTN general-purpose vibration sensor was mounted at the top of the housing of the rolling-element bearing closest to the disc, where imbalance was introduced. Vibration signals were recorded using a 24-bit AVM4000^®^ data collection unit. Analyzed data consisted of 235 signals, each of 10 s length, with a sampling rate equal to 25 kHz (AVM4000 supports 25, 50, and 100 kHz). The total number of points analyzed was equal to 235 [signals] × 10 [seconds] × 25,000 [samples/second] = 58,750,000 data points with double precision. Individual signals were collected periodically, ca. every minute.

### 3.4. Characteristic Imbalance Component

In the investigated example, output shaft experiences gradually increased imbalance. The nominal speed of the machine was 3000 RPM (50 Hz), and the transmission ratio was 23/67, so the nominal frequency of the unbalanced output shaft was 50 Hz × 23/67 = 17.16 Hz. The current paper investigates output shaft component for two versions of an angular signal, namely when the signal is resampled with respect to the reference (healthy) input shaft, and when the signal is resampled with respect to the faulty output shaft. Notably, when a signal is resampled with different ratios, its resampling parameters vary, which results in differently ordered spectrum parameters. Details of spectral parameters are illustrated in Table 1. Note that Table 1 demonstrates the process; thus, certain numerical roundings were applied according to corresponding analytical considerations.

Column one in Table 1 defines the analyzed parameter. Column two refers to regular frequency spectrum data. The last two columns refer to the signal resampled with respect to the input shaft and output shaft, respectively. The nominal length of the signal in each case is the same and is equal to 10 s. The sampling frequency is not applicable to resampled data. The nominal number of samples is the same, which means that during the resampling process, the new number per samples per rotation is calculated as the average number of samples in rotation. In practice, in the case of a resampled signal, non-integer cycles are trimmed (here, less than 1%). The nominal speed of the input shaft is 3000 RPM (50 Hz), while the nominal speed of the output shaft is smaller by a factor of 23/67. Since the input shaft rotates 50 times in one second, it rotates 10 times more in 10 s. Analogously, the output shaft rotates 10 times more than in one second in each signal. The number of samples per rotation describes how many data points are nominally collected in each shaft rotation. Since the input shaft rotates faster, the number of samples per rotation is smaller. For individual shafts, the number of samples per rotation is calculated by dividing the total number of samples by the number of rotations. The same number of input shaft rotations and average number of samples per rotation are a coincidence resulting from the length of the signal, sampling frequency, and shaft speed. The individual number of samples per rotation limits the order bandwidth to its half. At the same time, the spectral resolution is the reciprocal of the number of rotations of each shaft, and is different for an order spectrum. Finally, as indicated in Table 1, although represented by the same spectral index, spectral components corresponding to individual shafts have different orders on order spectrums calculated with respect to the input shaft and the order spectrum calculated with respect to the output shaft.

## 4. Experiment Results

The presented study compares the ability of tracking the increasing imbalance condition of the output shaft among different trend definitions. As described in detail, the problem is that the speed of the output shaft under investigation is related to the measure speed by a rational number, precisely by 23/67. For this reason, the question arises whether it is sufficient to calculate the output shaft imbalance component from the order spectrum calculated with respect to the reference input shaft, or whether it is necessary to apply an additional signal processing algorithm to resample the signal with respect to the output shaft.

### 4.1. Trend Analysis

The analysis presented in this paper covers four different types of HIs, which are shown in Figure 2. Thus, the paper compares the proposed result with current practices, namely PP, RMS, VRMS, the shaft harmonic in the frequency domain and shaft harmonic in the order domain without proposed rational resampling. Comparative results are presented both qualitatively as well as quantitatively. All spectral HIs were calculated from full-resolution frequency/order spectra. If not stated otherwise, HIs were calculated from acceleration signals. Narrowband HIs were calculated as spectral peak values, that is, the maximum of spectral amplitudes from a specific bandwidth, here related to an output shaft. The complete list of investigated scalar indicators is given below:

- PP: peak-to-peak value, calculated in time domain;
- RMS: root mean square value, calculated in time domain;
- VRMS: velocity RMS, calculated in time domain, ISO 20816 (10–1000 Hz);
- Output_Shaft_x1: output shaft component in frequency spectrum;
- Output_Shaft_x1_IN: output shaft component in order spectrum calculated with respect to reference input shaft;
- Output_Shaft_x1_OUT: output shaft component in order spectrum calculated with respect to output shaft.

**Figure 9:**
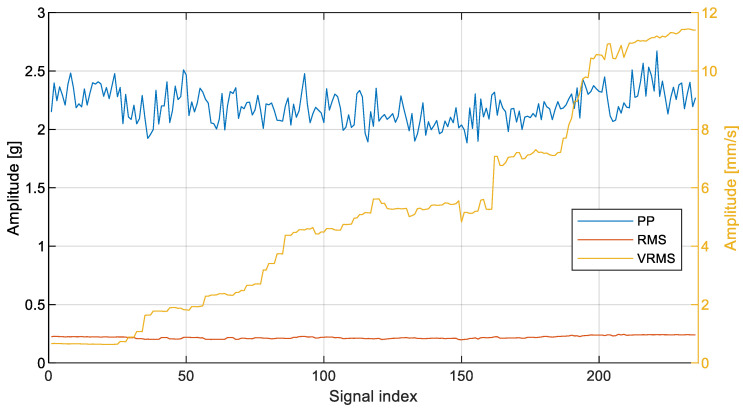
Broadband and narrowband HIs in detection of imbalance state. Signal index represents subsequent instances of vibration signals.

**Figure 10:**
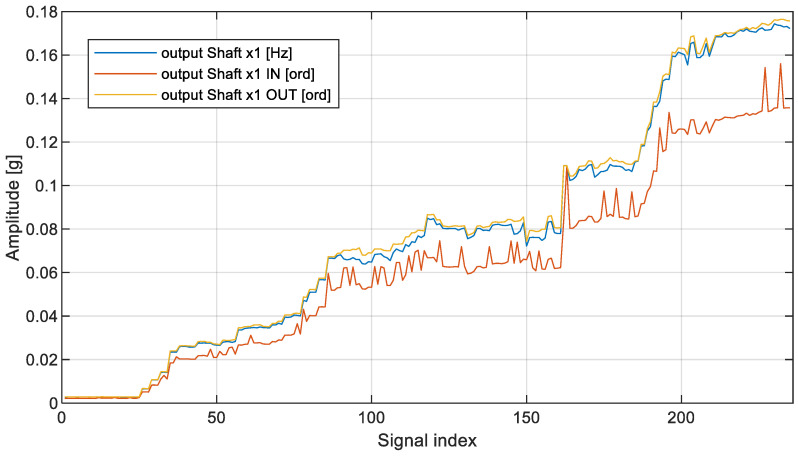
Narrowband output_shaft_x1 HIs in detection of imbalance state. Abscissa is the same as in Figure 9.

Figure 9 illustrates two broadband PP and RMS indicators along with the wideband VRMS indicator, while Figure 10 illustrates the trend of the three remaining narrowband spectral indicators.

Figure 9 shows that PP and RMS estimators were not able to indicate the development of the output shaft imbalance state. The VRMS estimator, on the other hand, clearly shows the rise of centrifugal force caused by imbalance. According to ISO 20816, the initial VRMS level (ca. 1 mm/s) refers to a new machine condition, while the final VRMS value (ca. 11.5 mm/s) is not permissible. However, the VRMS indicator is limited to fault detection only, and is not suitable for fault identification, which is critically important in the case of shaft imbalance. In the case of narrowband HIs, according to the Refs. [23,24], it is the relative increase which defines the severity of the fault, namely, a serious increase occurs for an 8 dB increase (factor of 2.5), whereas the alarm value refers to a 20 dB increase (factor of 10).

Description of labels for Figure 10 are as follows:

▪ Output Shaft x1 [Hz]—classical approach without resampling;
▪ Output Shaft x1 IN [ord]—classical approach with classical resampling;
▪ Output Shaft x1 OUT [ord]—classical approach with new proposed resampling.

In the case of narrowband analysis, the results were, to some extent, unexpected. The first trend data series named “output Shaft x1” was surprisingly very close to the trend calculated from the signal resampled with respect to the output shaft, which requires additional signal processing transformations. The second data series, which follows a classical resampling scenario, that is, where the signal was resampled according to the reference shaft (here input shaft), was still able to detect very significant changes, though at the cost of evidently larger variance.

### 4.2. Quantitative Assessment of Indicators

For quantitative assessment of individual narrowband health indicators, two measures are introduced, namely, the relative difference *RD* between last and first value (Formula (1)) as well as variance *var* calculated with respect to a moving average value with window length *k* equal to 5 (ca. typical 2% of number of elements *N* in data set), given by Formula (2). Formula (1) was derived form a common standard used for threshold calculations.

(1) $$RD=\frac{x_{N}}{x_{1}}\cdot 100\%$$

(2) $$var=\sum_{n=1}^{N}\left(x_{n}-\frac{1}{k}\sum_{i=n-k+1}^{n}x_{i}\right)$$

**Figure 11:**
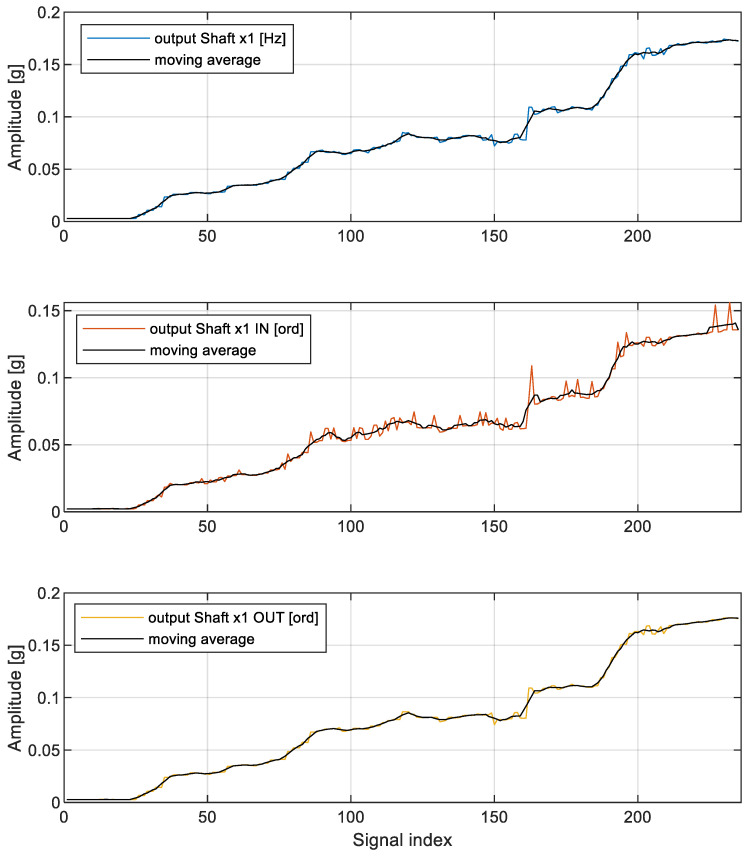
Graphical illustration of moving average value for individual scalar health indicators. Abscissa is the same as in Figure 9 and Figure 10.

Figure 11 illustrates the moving average along with each narrowband estimator. The quantitative assessment of individual data is illustrated in Table 2.

By comparing variances of data in Table 2, it might be concluded that the variance of narrowband output shaft order calculated with respect to the output shaft is nearly 17% smaller than the variance of the narrowband output shaft characteristic frequency calculated directly in the frequency domain and, at the same time, 75% smaller than the variance of the narrowband output shaft order calculated with respect to the reference input shaft (as typically met in industrial applications). A further investigation of the shape of the imbalance component in analyzed spectral representations points to a conclusion that order representation of spectral components might be undesired when performed with respect to a shaft with a rational transmission ratio, as is the case of the conducted experiment. As shown in Table 2, when resampling is performed with respect to the shaft of interest, the relative increase of shaft components is largely possible, and at the same time, the trend time series is characterized by the smallest possible variance, making technical diagnostics more reliable.

### 4.3. Analytical Considerations

**Figure 12:**
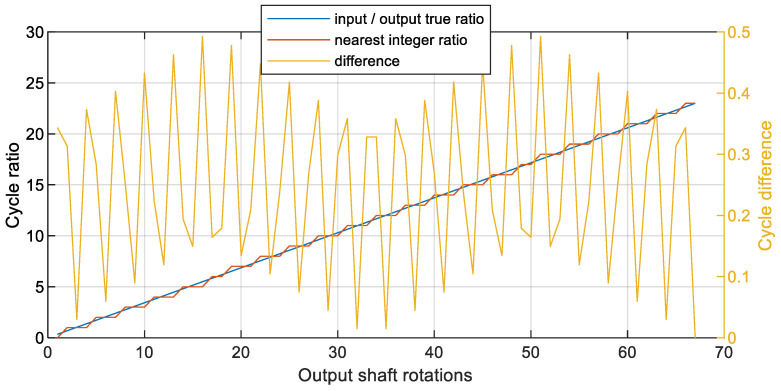
Ratios of cycles for the first 67 rotations of the output shaft.

From the quantitative analysis of the variance, it is shown that for rational transmission rations, it is recommended to perform resampling with these transmission ratios included in the algorithm. The explanation for this observation result is additionally given in Figure 12.

Figure 12 illustrates three sets of data for the first 67 rotations of the output shaft (since the gearbox ratio is z1/z2, where z1—number of teeth on pinion, z2—number of teeth on gear). The first data series were calculated as [z1/z2]*[1:z2], representing the true ratio of the output shaft cycle with respect to the input shaft. Since the shafts were phase-locked, the data were monotonically increasing. The second data series is an auxiliary one, and was needed to calculate the third data series. The second data series represents the nearest integer to the data series 1. The third data series represents the absolute value of the difference between the two. In this way, it represents how close the cycle of the output shaft was to any integer multiple of the input shaft. As illustrated in Figure 12, this relation is composed of three linear functions, including local peak-to-peak values, mirror-like translation, and modulation. Most importantly, as clearly seen from Figure 12, the perfect match to an integer ratio occurs every 67th rotation of the slow shaft. According to the hunting tooth theory [25,26], this is because both numbers of teeth are prime numbers.

The presented analytical considerations show how to assess the degree of potential improvement when resampling is done using rational transmission ratios for shafts without direct phase marker measurement. In practice, this concept is performed by modifying the number of points of the new abscissa axis (angle domain) before the first interpolation by multiplying the number of rotations and the new number of samples per rotation additionally by the rational ratio between the reference shaft and shaft of interest (in the paper by z1/z2).

## 5. Conclusions

Condition monitoring systems of rotating machinery are preferably equipped with a speed sensor, typically a 1-per-rotation phase marker mounted on the driving (fastest) motor. However, for some applications, like belt transmissions, systems use multiple PM signals, typically to monitor the slip. The current paper shows, both qualitatively (Figure 9 and Figure 10) as well as quantitatively (Table 2), that for drive trains with a rational transmission ratio, it is possible to enhance the detection and identification of an increase in phase-locked components using an additional step within the process of resampling. As a result, it is an alternative solution to (and cheaper than) using a separate PM sensor. This is especially important in industrial installations, where multiple vibration channels use a single PM signal (the most commonly used scenario).

Successful identification of faults of rotating machinery requires a priori definition of mathematical formulas (also called signal processing paths) of narrowband scalar features. In the case of identification of shaft imbalance, this definition refers to shaft frequency, or more precisely, to the shaft order, which defines the location of component of interest on the spectral axis. As shown in the paper, this relatively simple scenario becomes more complicated for machinery where the imbalance occurs on the shaft different from the one on which the speed is measured, and most importantly, on such a shaft, the speed of which is related to the reference shaft by a rational number. As a result of the conducted experiment and analysis, the paper gave two added values to this field of technical diagnostics. Firstly, the paper showed that by processing signal resampling with respect to the shaft of interest, the trend analysis might significantly improve in terms of its dynamics and variance. Secondly, the paper showed that for rational transmission ratios, resampling might be slightly detrimental to trend analysis, especially when the average number of samples per rotation for the reference shaft is significantly different from the average number of samples per rotation for the shaft of interest even if the overall spectral peak amplitudes might be similar, and the variance of the trend points is significantly larger (here 75%). This observation might be overlooked during diagnostics analysis of a single signal, but it is important for long-term condition monitoring. As shown in the analytical considerations, this is especially important when the transmission ratios are expressed with prime numbers. Although such solutions are desired from the gearbox durability point of view, the paper showed that they raise an additional challenge for vibration-based condition monitoring of subsequent phase-locked components of drivetrains.

## Figures and Tables

**Table 1:** Selected spectral parameters.

Parameter	Frequency Spectrum	Resampling Input Shaft	Resampling Output Shaft
Nominal length	10 s	10 s	10 s
Sampling frequency	25,000 Hz	-	-
Number of samples	250,000	N = 250,000	N = 250,000
Input shaft nominal speed	50 Hz	-	-
Output shaft nominal speed	50 × 23/67=17.16 Hz	-	-
No. input shaft rotations	-	Nrot = 50 × 10 = 500	-
No. output shaft rotations	-	-	Nrot = 17.16 × 10 = 171
No. of samples per shaft rotation	-	Nsamp = N/Nrot = 500	Nsamp = N/Nrot = 1461
Spectral range	12,500 Hz	Nsamp/2 = 250 [ord]	Nsamp/2 = 730.5 [ord]
Spectral resolution	0.1 Hz	1/Nrot = 1/500 = 0.002	1/Nrot = 1/171 = 0.00585
Spectral order–input shaft	-	1	2.91
Spectral order–output shaft1		0.343	1
Input shaft spectral index	500	1/0.002 = 500	2.91/0.00585 = 500
Output shaft spectral index	171	0.343/0.002 = 171	1/0.00585 = 171

**Table 2:** Quantitative assessment of narrowband health indicators.

Indicator	Amplitude RD	Variance
output_Shaft_x1 [Hz]	625.5%	0.00109
output_Shaft_x1_IN [ord]	625.3%	0.00366
output_Shaft_x1_OUT [ord]	629.7%	0.00091

## Data Availability

Not applicable.
